# International Medical Congress

**Published:** 1885-10-20

**Authors:** 


### INTERNATIONAL MEDICAL CONGRESS.

The commotion in regard to the new committee for organization of the Medical Congress, after eliminating the incongruous materials, must eventually settle down upon a satisfactory basis. Agitation and discussion in a professional democracy, as in a political republic, result in good to all concerned. Like fermentation in wine, they work off the impurities, and impart strength and bouquet to the whole. The attitude of the medical press in the United States and in England towards the issue raised in regard to the International Congress is calculated to bring out the facts connected with the "Situation." It is highly important that no reserve shall be maintained by those withholding their influence from the Congress, as to the grounds of their withdrawal from the new organization; and the indefiniteness of the preamble and resolutions published thus far by those refusing to undertake the duties assigned them leaves the medical public in doubt as to the real cause of their action.

The personal relations of certain individuals to the work at present may have influenced their retirement, to an extent that no convictions of duty to the medical profession could influence them to resume any sort of relations to the new committee of organization. It must, therefore, be taken as a foregone conclusion that those who have withdrawn will remain out of the International Congress, and, further, that they will throw all the hindrances possible in the way of the success of the plan of the American Medical Association to harmonize the action of the different elements of the profession in this country with those in foreign countries, which are expected to co-operate in securing a satisfactory result. No one who has read the communications of Sir James Paget and of Sir William McCormack can doubt that they have been prompted to present their views on the subject of organizing the Congress by parties in the United States who are disaffected, and that Dr. Henry D. Noyes should have elicited from Dr. Hansen-Grut an *ex parte* statement is not to be viewed in any other light than simply an acquiescence in his avowed opposition to the new programme. There has not only been an alliance between the disaffected amongst us, but a league with those abroad, to defeat the independent movement upon a liberal basis of recognition for the rank and file of the Medical profession in America. How far this narrow-minded policy can succeed in poisoning the minds of high-toned gentlemen, who are sincerely interested in promoting scientific investigations by an international reunion, in 1887, at Washington City, must depend upon factors in the equation which are not yet well defined. For the proper solution of the problem, the unknown quantity of Common Sense must have an important bearing; and it would seem that, after all the agitation of the matter amongst ourselves, nothing has been done except to settle down upon the ways and means of accomplishing what pertains to the American element, in which, as far as appears to us, the foreign elements have no lot or part. Other countries adopted their own domestic arrangements preparatory to holding meetings of the Congress in their respective localities, without any interference from the outside world, and we can be allowed some indulgence of our fussy mode of doing business upon the general principle of "All's well that ends well." Our people are not such a set of boobies as to need the dictation of others in the management of these private matters, and the result will prove their wisdom and discretion.

